# Stress and the Multiple-Role Woman: Taking a Closer Look at the “Superwoman”

**DOI:** 10.1371/journal.pone.0120952

**Published:** 2015-03-27

**Authors:** Monika K. Sumra, Michael A. Schillaci

**Affiliations:** Department of Anthropology, University of Toronto Scarborough, Toronto, ON, M1C 1A4, Canada

## Abstract

In the academic literature there is debate as to whether women who engage in multiple social roles experience more or less stress than women in fewer roles. For the present research we examined the relationship between levels of engagement in seven distinct roles and perceived stress and life satisfaction in a small non-random sample of women in North America (N = 308). We did not find a significant correlation between role engagement and perceived stress, though we did find a small but significant positive correlation between role engagement and life satisfaction. Similarly, in a subset of the participants (N = 31), there was not a significant relationship between the level of role engagement and physiological stress as measured by hair or urinary cortisol levels. We found a significant negative correlation between perceived stress and life satisfaction, and role satisfaction. The results from multiple regression models did not identify the level of role engagement as a significant predictor of either perceived stress or life satisfaction. Role satisfaction in addition to several life style variables such as the frequency of sex and exercise were identified as significant predictors of both outcome variables. We also examined the popularized notion of the “superwoman”, which we defined as women who fell within the 4^th^ quartile of role engagement, or those engaged in the wife/mother/worker/homemaker role combination. Based on popular discourses surrounding the superwoman we expected that superwomen would exhibit higher levels of perceived stress. Our results revealed that superwomen do not experience a significantly higher level of perceived stress than non-superwomen. The results of our study therefore suggest that multiple role engagement in women, even at a relatively high level as experienced by “superwomen”, is not associated with significantly higher stress, or reduced life satisfaction.

## INTRODUCTION

As women continue to balance working outside the home with their domestic responsibilities, the idea of engaging in multiple roles simultaneously, or “doing it all”, has become a valued social norm [1,2]. In both popular and academic discourses on the “multiple-role woman”, stress is often considered an inevitable outcome. Stress has been described as a heightened state of emotional or physical arousal occurring when demands from the environment, such as engaging in multiple roles, place pressure on an individual’s capacity to adapt [3]. Although small bouts of stress have been shown to be protective and advantageous to health [4], chronic or prolonged stress elicits adverse physiological responses such as increased blood pressure, compromised immune system, inflammation, and diabetes [5], all significant risk factors for coronary heart disease (CHD). For example, Janssen et al. [6] highlighted the need to understand how the number of roles and role satisfaction may influence the occurrence of CHD among women. The authors examined multiple roles in relation to subclinical CHD as measured by the progression of coronary artery calcification [6]. Results from that study revealed that rewarding or satisfying roles are protective psychosocial factors that may mitigate the progression of arterial calcium build up in black women [6]. There is also evidence that indicates women who perform multiple concurrent roles often put the needs of others (i.e. spouse, family members, and employer) before their own [7, 8]. This may contribute to women ignoring potential contributors to CHD such as increased stress or even result in delayed treatment [9].

The corticosteroid cortisol is an important end product of one of the body’s major stress response systems, the hypothalamic-pituitary-adrenal-axis (HPAA). Cortisol measured in saliva, serum, urine and hair can be used as a biomarker to assess endogenous cortisol secretion associated with clinical conditions such as severe stress, depression and other systemic diseases (i.e. Cushing’s syndrome) [10]. Saliva, serum and urine are the most commonly utilized biological matrices to measure cortisol levels [11]. Saliva, serum and urine are subject to circadian variation in cortisol secretion resulting in fluctuating levels over a 24-hour period [12]. Salivary, serum and urinary cortisol levels are typically measured over a 24-hour period usually starting with a first morning sample with subsequent samples taken at intervals throughout the day. Single urine samples collected at any given time reflect cortisol secretion since the last urinary void. Only urine collected throughout the day captures cortisol secretion over a 24-hour period [13], [14]. Saliva and serum are considered to be “point estimates” for cortisol secreted prior to collection (~20 minutes for saliva and <3 minutes for serum). Cortisol concentration in hair, on the other hand, has been described as a novel biological marker for measuring stress over a longer period of time, with reliable and validated tests to measure cortisol levels in hair becoming available in recent years [15]. Uptake of cortisol in scalp hair occurs primarily through blood circulation when the hair shaft is formed [16]. It is argued that because of the balance between serum levels and hair cortisol concentration, and because hair grows on the scalp at an average rate of 1cm per month [17], measurements of endogenously produced cortisol in the hair may reflect average hormone levels over a period of weeks or months [10, 18, 19]. Recent research on multiple stressors and physiological stress has revealed that the level of the stress hormone cortisol in hair is a predictive biomarker for cumulative or chronic stress in men [20] and pregnant women [21]. Additionally, in pregnant women, hair cortisol levels have been positively correlated with self-perceptions of psychological stress [21]. Research has also demonstrated that increases in both self-reported and physiological stress occur in women as engagement in a specific role intensifies [22].

Although role-related stress in women has been discussed in the academic literature [6], [11, 21, 23–51] whether stress is an outcome of multiple-role engagement and, if so, to what extent women experience it remains unclear. For example, Pearlin [47] reported that married employed women who are overloaded with housework are more likely to experience an increase in psychological stress. More recently, Maclean, Glynn and Ansara [44] examined two self-reported measures of stress. Their study evaluated the relationship between the roles of mother (single/partnered) and worker (employed/unemployed) on psychological or perceived stress among 7364 North American women. In that study, single unemployed mothers were twice as likely to report high levels of stress than all other groups. Single mothers whether employed or not, were most likely to report higher levels of stress and feelings of being overloaded [44]. Using Statistics Canada’s 2000 Canadian Community and Health Survey (CCHS) 1.1, Muhammad and Gagnon [44] found that divorced women living alone had a 3.23 times higher odds of declaring higher stress than married women living with their partners, with or without children.

Stress research on multiple-role engagement and women has focused primarily on the interaction of two specific role categories, the worker/employee and familial roles [33, 38, 41, 46, 49, 50, 52, 53]. What is missing from the literature, however, is consideration of the variability and complexity of roles that exist within the family role category. Although roles such as caregiver, homemaker and/or wife have been included under the umbrella of the “family role”, these roles may within their own right contribute to the degree and intensity of multiple-role engagement, consequently influencing the level of stress and any associated adverse psychological and/or physical health outcomes [34, 53–55]. As such, these components should be regarded as roles in and of themselves.

Research on the relationship between multiple roles and role/life satisfaction offer mixed results. Some studies report greater overall satisfaction associated with multiple-role engagement [32, 56, 57], while others report lower satisfaction [58, 59]. Tiedje et al. [60] reported that women who engaged in multiple roles did not always exhibit increased perceptions of enrichment or satisfaction. Reid and Hardy [61] found that although the number of roles was associated with psychological distress among women, once role satisfaction was accounted for, the number of roles had no effect. Increased social capital in the form of supportive actions of others (such as advice and reassurance) has been described as effective in mitigating psychological stress [62–65]. Similarly, there is a significant correlation between lack of social support and physiological stress as measured by serum cortisol [11].

### Role theory: Enrichment versus depletion

What might contribute to the inconsistent findings in the research on stress among women? Two competing perspectives within role theory have been proposed to explain the effects of multiple roles on psychological stress/distress. The role-strain/overload perspective, also referred to as the depletion or scarcity hypothesis (hereafter the Depletion Hypothesis), proposes that an increased number of roles leads to overload and strain [32]. Among multiple-role women, psychological stress has been related to individual role stress, as well as stressful experiences across roles [65]. Additionally, this hypothesis suggests that maintenance across multiple roles can create stressful conflict between or among roles resulting in dissatisfaction with one’s ability to perform these roles [49, 60]. This can result in adverse psychological and physiological outcomes [32]. Alternatively, the role enhancement perspective, also referred to as the role accumulation, expansion or enrichment hypothesis (hereafter the Enrichment Hypothesis), suggests that multiple-role engagement enhances an individual’s resources, social connections, power, prestige and emotional gratification [32, 66–68]. The Enrichment Hypothesis suggests that multiple roles can actually serve as a buffer against stress [49], and that feelings of well being generated in one role can positively influence experiences in other roles. See [49, 69, 70]. This hypothesis reflects research that has shown that engagement in multiple roles such as caregiver (of a parent), mother, wife and employee, is associated with better psychological well-being and reduced stress [71].

### The “Superwoman” as an archetype of female identity

Female identity is one aspect of social identity that refers to the meaning women attach to their membership in the category “female” [63]. In the West, engagement in multiple roles has been regarded as a key characteristic of female identity [1, 2]. Perhaps not surprisingly, discourses surrounding the social construction of female identity are well developed. Constructs such as the “alpha female”, “career woman”, “soccer mom”, “domestic goddess” and even “slut” are well-established female archetypes. Whether women actually psychologically or physiologically experience these identities, however, has not been well explored. One construct of female identity that has received much attention in both the popular media and academic literature is the “superwoman”. The superwoman identity refers to a woman, who performs a combination of multiple concurrent fulltime roles such as wife, mother, worker, homemaker and caregiver [72, 73]. In the academic literature, “superwomen” are often said to be “double-burdened” with the responsibility of fulfilling both their domestic and career obligations [30, 74–76]. Psychological and physiological stress among such women is often regarded as a consequence of engaging in multiple social roles. The term “Superwoman Syndrome” has been used to describe this experience of stress in multiple-role women [77] resulting from the pressure associated with achieving perfection in the performance of these roles [35, 64, 78, 79]. Within this context, stress has also been described as a consequence of role conflict [49, 80, 81].

Surrounding the popular notion of the superwoman is the idea that she is a woman who “does it all, and does it well”. She is often portrayed as successfully balancing her domestic and career responsibilities. First proposed by Catherine Steiner-Adair in 1986 [72], the Super Woman Construct (SWC) was developed to examine the development of eating disorders as an outcome of the stress associated with internalizing the superwoman ideology. For her study Steiner-Adair [72] interviewed a group of 32 adolescent girls aged 14 to 18 from a private girls’ school in upstate New York. Through these interviews two patterns of responses were defined *a posteriori*, one of which was termed the “superwoman pattern”. According to Steiner-Adair [72] those who responded in the superwoman pattern, 1) adhered to traditional female values of caring and sensitivity, along with values associated with being “self-made” such as independence, autonomy, and success, 2) identified the “independent and autonomous successful superwoman as society’s ideal image of a woman, and 3) self-identified with the image of the superwoman as their own ideal image of a woman. The results of the semi-structured 20-minute interviews revealed that 40% of the respondents (n = 12) ascribed to the SWC. Additionally, these respondents also scored highly for vulnerability to eating disorders as measured by the Eating Attitudes Test or EAT [72].

In a similar study, Thornton, Leo and Alberg [82] describe the superwoman ideal as consisting of four components; 1) increased concern with physical appearance, 2) heightened interest in maintaining satisfactory interpersonal relationships, 3) striving to maintain a level of independent achievement, and 4) successful performance across many diverse roles. Women who conformed strongly to the superwoman ideal were found to be at greater risk of having eating disorders [82]. With respect to diversity of roles, Timko et al. [83] noted that the greater number of roles a woman deemed as important to their sense of self, the greater the risk of eating disorders.

Defined as a woman who “wants to have it all” by “excelling in both traditional feminine and masculine roles” [84], the “superwoman hypothesis” has also been used in research to examine how the stress of performing multiple concurrent roles is associated with eating disorders. This hypothesis describes the superwoman as a woman who is stressed due her simultaneous efforts to be thin and attractive, a loving wife and mother, and, a strong independent woman [84].

Other researchers have described the superwoman as possessing specific characteristics or traits. According to Woods-Giscombé [73] the “Superwoman role” has been regarded as a social phenomenon influencing the ways in which African-American women experience and report stress. In that study, Woods-Giscombé [73] described specific characteristics associated with what she calls “the Superwoman Schema (SWS)”. These characteristics include a woman’s obligation to manifest strength, obligations to suppress emotions, and obligation to help others [73]. Contextual factors such as history of racial stereotyping, lessons from mothers and spiritual values also inform Woods-Giscombé’s [73] SWS. In addition, Woods-Giscombé [73] found that women associated both benefits and liabilities with the superwoman ideology. Benefits included preservation of self and family or community, and liabilities included relationship strain, emotional eating, poor sleep, and embodiment of stress (anxiety, depression, poor mental health). Similarly, other researchers have suggested that multiple-role experiences may lower levels of stress as women become more organized in managing their multiple roles [25, 85].

### The Superwoman: Enrichment or Depletion?

It is evident from the previous research just described that there is uncertainty as to whether “superwomen” are more or less stressed than non-superwomen. While many researchers have associated stress as an adverse health outcome of engaging in multiple roles [1, 2, 8, 29, 32, 35], others have suggested that being a superwoman is not necessarily maladaptive, and that engagement in multiple roles may reduce or mitigate stress [61, 73]. Despite public and academic interest in stress and the superwoman construct, empirical research in this area is limited. Specifically, research on how stress is experienced by superwomen engaged in multiple roles is not well developed. The present study addresses this by examining the superwoman as an archetype of the multiple-role woman.

The Depletion and Enrichment Hypotheses of role theory present an interesting framework within which to examine the superwoman ideology, multiple-role engagement, and women’s perceived and physiological stress. For the present research we test the Depletion and Enrichment Hypotheses by examining the relationship between levels of engagement in seven distinct roles and two outcome variables: stress (perceived and physiological) and life satisfaction in a sample of North American women. If the Depletion Hypothesis is correct, we predict women engaged in more roles will experience higher levels of perceived and physiological stress and lower levels of life satisfaction than those women engaged in fewer roles. If, on the other hand, the Enrichment Hypothesis is correct, we expect women engaged in more roles to experience lower stress, and higher levels of life satisfaction than women engaged in fewer roles.

## METHODS

### Participant Recruitment and Exclusion

The present study was carried out from April 2012 to June 2013. A questionnaire was designed and made available to women aged 18 and older on the website surveymonkey.com (S1 File). The link to this survey was posted on various women’s health and wellness websites. The survey comprised 85 questions designed to assess perceptions of stress, social capital, and life satisfaction, as well as to collect demographic information such as income, age group, education and hours of employment.

Data on engagement in role categories specifically identified in our review of the academic literature were collected. This review revealed that 7 role categories contribute both to the intensity of multiple-role engagement, and to the degree in which this intensity is reflected in perceived and/or physiological stress among women. These roles include wife, mother, student, worker/employee, homemaker, volunteer and caregiver for parent(s), family members, or a special needs child [21, 23, 27, 28, 29, 51, 52, 69, 86, 87]. Data on the frequency of exercise (per week) and sex (per month) were also collected from the survey responses. Respondents were asked if they would like to participate in the second phase of the study, which involved collection of personal information and biological samples (urine and hair) for the measurement of cortisol.

Of the 440 surveys obtained 132 participants were excluded from the dataset. Participants were excluded if surveys were incomplete/had missing information, and/or if they reported engagement in “0” roles. Participants in the biological sampling group were excluded if they reported being on a form of chemical birth control, or they reported being menopausal as designated by a physician. The remaining 308 participants included both the 277 women who participated in the survey phase of our study (the survey-only group), and the 31 women who in addition to the survey phase, also participated in the biological sampling phase (biological sampling group) of our study. Role engagement was calculated as fractionated roles to accommodate varying degrees of involvement in specific roles (e.g. worker role based on number of average number of hours worked in a given week).

### Perceived Stress Scale (PSS), Social Capital, and Life Satisfaction Measures

The Perceived Stress Scale (PSS-10) [88] was used as an index of perceived psychosocial stress, with higher PSS scores reflecting higher self-reported psychological distress. The PSS is perhaps the most widely used and validated psychological instrument for measuring the perception of stress (see [20,21, 45, 89–91]), and has been shown to be an effective measure of perceived chronic stress with an acceptable level of internal consistency (Cronbach’s α >0.70) [92]. The questions of the PSS are designed to ascertain how unpredictable, uncontrollable, and overloaded respondents find their lives. The scale also includes a number of direct questions about current levels of stress experienced. The questions are general and relatively free of content that may be specific to any subpopulation. The PSS questionnaire was administered a second time to each of the 31 women in the biological sampling group upon collection of the final urine sample. A t-test comparison of pre- (Mean = 18.84, SD = 5.57) and post- (Mean = 17.87, SD = 6.05) sample collection scores was not significant (t = 1.115, P = 0.274). For this group, PSS scores were, therefore, calculated as an average of both the pre- and post- sample collection scores.

This study also included an assessment of life satisfaction. A single question was asked, “Describe the level of satisfaction and fulfillment you feel in your life”. Respondents were asked to indicate their response on a 5-point Likert scale (0-none, 1-low, 2-moderate, 3-high, and 4-extemely high). Cohen’s Social Network Index (SNI) [62] was used as a measure social capital. Cohen et al.’s [62] work on social ties and susceptibility to the common cold revealed that individuals with more diverse social networks had greater resistance to upper respiratory illness, even without considering the nature of those social relationships. The SNI is a validated measure of network diversity and network size with an acceptable level of internal consistency (Cronbach’s α = 0.77) [93]. Network diversity is defined as the number of social roles in which the respondent has regular contact. The maximum number of high-contacts is 12 and includes: spouse, parent, child, in-law, close relative, close friend, religious group member, student, employee, neighbor, volunteer and other group member. Network size is defined as the total number of people with whom the respondent is in regular contact. Both network diversity and network size are based upon having contact at least once every two weeks [62].

### Urinary Cortisol

As this study examined urinary cortisol secretion among women, it was necessary to account for the reproductive cycle, which is associated with HPAA functioning [13]. Fluctuations in cortisol levels during the menstrual cycle have been reported [94]. To account for this fluctuation, and intra- and inter-individual variability in cortisol levels due to other non-measured factors, Nepomnaschy et al. [14] suggest that a minimum of 10 first-morning urine specimens over a given month are needed. First morning urine provides an integrated measure of overnight cortisol secretion. This method allows for an evaluation on an individual’s overall physiologic stress across several days. Overnight urinary cortisol is considered preferable, as it is less likely to be affected by any diurnal variation or diurnal confounders [13]. In the present study, we employed a 30-day sampling protocol rather than a single 24-hour sampling to account for cortisol fluctuation over a woman’s reproductive cycle [95]. Over the 30-day period, a total of 15 first-morning urine samples (one sample every other day), were self-collected by each participant in the biological sampling group. Participants were asked to store these samples in the freezer until their scheduled collection date. Frozen samples were collected weekly from participants and placed in a cooler to remain frozen during transport to the laboratory where they were stored in a freezer at −20 C. Urine samples were defrosted and aliquots (2 mL) were made from the original specimens and stored at −20 C until analysis. An aliquot of each sample was shipped on dry ice to the Maternal and Child Health Laboratory at Simon Fraser University in Burnaby, British Columbia, Canada. First Morning Urinary Cortisol (FMUC) was quantified using an enzyme-linked immunoassay for free cortisol (Quansys Biosciences, Logan, UT), validated in-house. Intra- and inter-assay CVs were 2.7% and 3.6%, respectively, and the lower limit of detection for urinary free cortisol was 0.5 ng/mL. To control for dilution effects, urinary free cortisol concentrations were corrected by specific gravity [96]. All samples were run in duplicate. All data points for which the coefficient of variation between duplicates was >12% were rerun. The mean urinary cortisol value (ng/mL) for each participant was calculated as the mean of the samples provided during the 30-day sampling period.

### Hair Cortisol

During the final week of urinary sample collection, each participant was asked to provide a sample of at least 25 strands of hair with an approximate length of 3 to 6 centimeters. Hair samples were sent to Viaguard/Accumetrics Inc. (Toronto, Canada) for laboratory analysis. Use of a special collection kit ensured the root ends faced the same direction. Participants were asked to cut hair as close to the scalp as possible and to ensure that the sample was collected between periods of dying hair, if hair was dyed on a regular basis. All kits were sealed and stored in individual envelopes. The first 3 centimeters of hair closest to the scalp were washed in isopropyl alcohol. Any hair follicles that were present were cut to ensure that only the cortisol from the hair shaft was extracted. Hair shaft samples were cut into small pieces with surgical scissors then weighed in a 1.5 ml tube. The weights of the samples ranged between 15–30 mg. Samples were then ground to a fine powder prior to an overnight extraction in methanol. The supernatant from the extraction was then removed and evaporated until completely dry. Once the methanol was removed, the sample was resuspended in 250ul of phosphate-buffered saline (PBS) at pH 8.0. Samples were vortexed for 1 minute until the extract was dissolved. The cortisol extracted from the hair was measured using DETECTX® KITS, Cortisol Enzyme Immunoassay Kit from Arbor Assays (Catalog #K003-H5W) as per the manufacturer’s directions with the reagents provided. The dissolved supernatant was compared to the kits’ cortisol standard curve and normalized with the weight of the hair to give pg/mg values.

### Defining the Superwoman

Superwomen seem to enact their identity in two distinct ways, either by 1) simply engaging in more than one role simultaneously, or 2) by striving to balance career and family life [1, 2, 8, 29, 31, 35, 61, 73]. While the former describes the number of roles a woman engages in, the latter considers a specific combination of roles. In order to examine this more closely it was therefore necessary to define the superwoman within the context of multiple-role engagement. In the present study superwomen were defined *a priori* as 1) multiple-role women within the fourth/upper quartile of role engagement (> 3.75 roles), and 2) women who simultaneously balanced their work and family life. Within our seven role categories, this was defined as women who actively engaged in the mother/worker/wife/ homemaker role combination.

### Debriefing: Sample and Setting

After the final collection of hair and urine samples, two 90-minute debriefing sessions were held. A total of 14 women attended. Preliminary survey and laboratory results were presented in a slideshow entitled “*Women and Stress Study 2012”*. Prior to the debriefing session a report was generated for each of the attendees. These individualized reports included, the participant number, hair cortisol level, average urinary cortisol level over the 30-day period, and analyses of surveys responses. These reports also included the averages for the biological sampling (N = 31) and survey-only (N = 277) groups so that each participant could get a sense of where their survey responses fell relative to the rest of the participants. Individualized reports were distributed at the end of the presentation and attendees were given the opportunity to ask questions. In addition to debriefing the participants, we took the opportunity to gain insight into the attendees’ narratives surrounding the superwoman ideology. Without any formal introduction about the superwoman concept, all attendees were asked if they would be interested in answering a few more questions related to the present study. Upon verbal agreement, each attendee was given a 3-question questionnaire. These questions were as follows: 1) “*Have you heard of the term superwoman*?, 2) “*What do you think it means to be a superwoman*?”, and 3) “*Do you consider yourself a superwoman*? *Why*, *or why not*?”. Participants were given 10 minutes to answer the questions. All 14 women completed the superwoman questionnaire. A discussion about the superwoman followed. During these discussions the principal researcher took notes. Though a formal textual analysis of the notes or the responses was not conducted, recurring references to specific terminology and themes that describe stress, multiple roles, and types of roles specific to the concept of the superwoman were recorded and interpreted

### Ethics Statement

This research, including our method of obtaining informed consent, was approved by the University of Toronto’s Research Ethics Board (Protocol #27117). Informed consent was obtained for each phase of our study from all participants. For the survey portion of this study, informed consent was provided electronically on the surveymonkey.com webpage. The webpage was designed such that participants could not proceed to the survey unless they clicked the “yes” option at the bottom of the consent letter describing the research. Signed consent forms were obtained from those participants providing biological samples before data collection, and were retained by the primary author.

### Statistical Analyses

Descriptive statistics including the mean, median and standard deviation were run for all variables to check for any data entry error and potential outliers. For our analysis of the superwoman construct we defined the “superwoman” both as women in the fourth quartile of role engagement (>3.75 roles), and women who simultaneously balance their work and family life actively by engaging in the mother/worker/wife/ homemaker role combination. All other women were defined as non-superwomen.

The differences between the biological sampling group and survey group were assessed using nonparametric Mann-Whitney U-tests. The correlation among variables was determined using the nonparametric Spearman’s correlation coefficient. After confirming there was not serial correlation or multicollinearity among variables, multiple regression analyses examining the relationship between our two dependent outcome variables, perceived stress and life satisfaction, and a series of predictor variables including age category, education, income, two measures of social capital (network diversity and network size), and various lifestyle variables were conducted. Differences in mean stress, satisfaction, social network diversity and social network size (measures of social capital) between superwomen and all other women, as defined by the fourth quartile of role engagement, were tested using a two-sample t-test after confirming the assumptions of data normality and equality of variances were satisfied. All statistical tests were conducted using Number Cruncher Statistical Systems (NCSS) statistical software package [97].

## RESULTS

Univariate statistics for 11 variables are presented in Table 1. Given that there were not significant differences between the survey (N = 277) and biological sampling groups (N = 31) across the primary variables, both groups were pooled (N = 308). Our pooled group of women reported participating in an average of 2.86 roles. Their mean reported network size was17.38 individuals. This represents the number of people in their social network with whom they communicate with at least once every 2 weeks. The mean Social Network Index (SNI) was 5.96. The average life satisfaction score was 2.52, indicating that women reported moderate to high life satisfaction.

**Table 1:** Results from univariate analyses for the survey group (SURVEY), biological group (BIO), and pooled group (POOLED).

		SURVEY			BIO				POOLED	
Variable	N	Mean	SD	N	Mean	SD	P	N	Mean	SD
^a^Age	277	3.574	1.601	31	3.677	1.351	0.634	308	3.584	1.576
^b^Education	277	3.169	1.089	31	2.806	1.223	0.158	308	3.133	1.106
^c^Combined Income	270	3.17	1.845	31	3.241	1.902	0.932	299	3.177	1.848
^d^Roles	277	2.861	1.201	31	2.896	1.287	0.921	308	2.864	1.208
^e^Life Satisfaction	276	2.511	0.755	31	2.613	0.844	0.491	307	2.521	0.764
^f^Role Satisfaction	276	2912	0.735	31	3.001	0.612	0.517	307	2.922	0.723
^g^Perceived Stress (PSS)	277	18.79	5.169	31	18.839	5.568	0.481	308	18.802	5.201
^h^Network Size (SNI)	277	17.299	8.559	31	18.129	7.182	0.325	306	17.383	8.424
^i^Network Diversity (SNI)	275	5.971	1.729	31	5.839	1.791	0.878	306	5.958	1.733
^j^Sex Frequency	254	6.35	7.519	29	5.34	6.65	0.473	283	6.247	7.43
^k^Exercise Frequency	275	8.116	10.781	31	6.871	7.032	0.972	306	7.99	10.46

Note: P-values associated with Mann-Whitney U-Test comparing mean values for the survey and biological sampling group

^a^Age Category 1 to 7 where 1 = 18–23 years, 2 = 24–29 years, 3 = 30–37 years, 4 = 38–44 years, 5 = 45–54 years, 6 = 55–64 years 7 = 65 years and over

^b^Education 1 to 5 where 1-high school, 2 = diploma from junior college or trade school, 3 = undergraduate university degree, 4 = masters degree, 5 = doctorate

^c^Combined income 1 to 6 where 1 = <41K/year, 2 = 41–70K/year, 3 = 70–100K/year, 4 = 100–130K/year, 5 = 130–160K/year, 6 = over 160K/year

^d^Roles—the number of roles a woman engages in (total possible roles 7)

^e^Life Satisfaction 0 to 4 where 0 = never, 1 = almost never, 2 = sometimes, 3 = fairly often, 4 = very often

^f^Role Satisfaction 0 to 4 where 0 = never, 1 = almost never, 2 = sometimes, 3 = fairly often, 4 = very often

^g^Perceived Stress (PSS-10)—The higher the PSS-10 score, the more likely it is that the individual will perceive that environmental demands exceed their ability to cope [89].

^h^Network Size (SNI)—defined as the total number of people with whom the respondent has regular contact with (i.e. at least once every two weeks)[62]

^i^Network Diversity (SNI)—defined as the number of social roles in which the respondent has regular contact with (i.e. at least once every two weeks) [62].

^j^Sex Frequency—number of times per month

^k^Exercise Frequency—number of hours per week.

The results from our nonparametric correlation analysis did not reveal a significant relationship between role engagement and perceived stress, but did reveal a significant correlation with life satisfaction (Table 2). In addition, perceived stress exhibited significant negative correlations with role satisfaction, combined income, age category, and frequency of exercise and sex. Life satisfaction exhibited significant positive correlations with role satisfaction, education, income, frequency of sex, and our two measures of social capital. Life satisfaction was significantly negatively correlated with perceived stress. Interesting relationships emerged for some specific variable combinations. For example, the relationships between the number of roles and age category, the number of roles and exercise frequency, and the number of roles and combined income were significant.

**Table 2:** Nonparametric Spearman correlations among variables used in the study. Correlations are listed below the diagonal, with probabilities (P-values) listed above the diagonal.

	Roles^a^	Life Satisfaction	RoleSatisfaction	PSS^b^	Network Size	Network Diversity	Education	Combined Income	Age^c^	Sex Frequency	Exercise Frequency
**Roles**	1	0.006	0.391	0.921	0.002	<0.001	0.221	<0.001	<0.001	0.069	<0.001
**Life Satisfaction**	0.156	1	<0.001	<0.001	<0.001	<0.001	0.024	<0.001	0.331	<0.001	0.135
**RoleSatisfaction**	0.049	0.527	1	0.151	0.001	0.008	0.639	0.051	0.010	<0.001	0.849
**PSS**	−0.006	−0.487	−0.414	1	0.081	0.151	0.187	0.007	0.020	0.012	<0.001
**Network Size**	0.171	0.231	0.197	−0.099	1	<0.001	0.533	0.241	0.848	0.678	0.136
**Network Diversity**	0.324	0.253	0.152	−0.082	0.724	1	0.840	0.075	0.216	0.005	0.942
**Education**	−0.070	0.129	0.023	−0.075	−0.036	−0.012	1	0.300	0.540	0.180	0.201
**Combined Income**	0.562	0.198	0.113	−0.157	0.068	0.104	0.06	1	<0.001	0.010	0.113
**Age**	0.485	0.056	0.146	−0.132	−0.011	0.071	0.035	0.491	1	0.009	0.317
**Sex Frequency**	0.108	0.343	0.214	−0.148	0.025	0.165	0.08	0.154	−0.155	1	0.118
**Exercise Frequency**	−0.300	0.086	0.011	−0.21	0.086	−0.004	0.073	−0.092	−0.057	−0.093	1

^a^Fractionated number of roles

^b^PSS, Perceived Stress Scale (PSS-10)

^c^Age category.

The results of the multiple regression models examining the predictors of perceived stress was significant (F = 8.34, df = 9, P<0.001, adj r^2^ = 0.194) (Table 3). Role satisfaction and exercise frequency were the only significant predictors of perceived stress. Of these significant predictors, role satisfaction was the most important, explaining approximately 15% of the variation in perceived stress. Our model examining life satisfaction was also significant (F = 13.93, df = 9, P<0.001, adj r^2^ = 0.299), identifying role satisfaction and frequency of sex as significant predictors of life satisfaction. Again, role satisfaction was the most important predictor, explaining 22% of the variation in life satisfaction.

**Table 3:** Results from the multiple regression analysis of perceived psychological stress (PSS) and life satisfaction for the pooled group.

**Dependent Variable: PSS**					
**Independent Variable**	**Regression Coefficient**	**Standard Error**	**Standardized Coefficient**	**P-value**	**Partial R** ^**2**^
Age Category	−0.303	0.23	−0.09	0.189	0.006
Combined Income	−0.326	0.196	−0.116	0.099	0.01
Education	−0.054	0.26	−0.011	0.836	0.001
Exercise Frequency	−0.066	0.028	−0.133	0.021	0.02
Roles	0.549	0.328	−0.128	0.096	0.01
Sex Frequency	−0.03	0.041	−0.043	0.469	0.002
Network Size	0.056	0.051	−0.089	0.275	0.004
Role Satisfaction	−2.78	0.4127	−0.396	<0.001	0.146
Network Diversity	−0.231	0.255	−0.076	0.366	0.003
**Dependent Variable: Life Satisfaction**					
**Independent Variable**	**Regression Coefficient**	**Standard Error**	**Standardized Coefficient**	**P-value**	**Partial R** ^**2**^
Age Category	−0.027	0.031	−0.055	0.388	0.003
Combined Income	0.032	0.026	0.08	0.221	0.006
Education	0.039	0.035	0.058	0.261	0.005
Exercise Frequency	0.004	0.004	0.061	0.257	0.005
Roles	0.044	0.044	0.072	0.317	0.004
Sex Frequency	0.011	0.005	0.113	0.04	0.016
Network Size	−0.002	0.007	−0.018	0.81	0
Role Satisfaction	0.481	0.055	0.477	<0.001	0.223
Network Diversity	0.037	0.034	0.086	0.275	0.004

The distribution of stress across role and role combinations is presented in Table 4. The role with the highest perceived stress was caregiver (N = 53, Mean = 20.377). The role of wife exhibited the lowest perceived stress (N = 185, Mean = 18.227). The difference in perceived stress between these two roles was significant (t = 2.150, P = 0.009). We also explored the distribution of perceived stress across role groupings that included the mother role. Single mothers (N = 26, Mean = 21.385) reported significantly (t = 2.903, P = 0.004) higher perceived stress than mothers with partners (N = 127, Mean = 18.134). The single mother/worker/homemaker combination had the highest perceived stress of all other mother combinations (N = 12, Mean = 22.67).

**Table 4:** Levels of perceived stress (PSS) for each of the seven roles and, and role combinations used in the study.

Role/Role Combination	N	Mean	SD
Wife 0	123	19.667	5.101
Wife 1	185	18.227	5.202
		t = 2.407, P = 0.017	
Mother 0	155	18.916	5.089
Mother 1	153	18.686	5.328
		t = 0.392, P = 0.695	
Caregiver 0	255	18.475	5.137
Caregiver 1	53	20.377	5.274
		t = −2.430, P = 0.017	
Student 0	198	18.742	5.413
Student 1	110	18.909	4.819
		t = −0.272, P = 0.786	
Worker 0	49	19.388	5.442
Worker 1	259	18.691	5.158
		t = 0.874, P = 0.380	
Volunteer 0	185	18.989	5.376
Volunteer 1	123	18.52	4.936
		t = 0.781, P = 0.435	
Homemaker 0	105	19.181	5.059
Homemaker 1	203	18.606	5.276
		t = 0.931, P = 0.353	
Mother with Partner	127	18.134	5.287
Single Mother	26	21.385	4.75
		t = 2.903, P = 0.004	
Mother X Homemaker	140	18.586	5.396
Mother X Worker X Homemaker	116	18.578	5.189
Single Mother X Worker	22	21.864	4.57
Mother with Partner X Worker X Homemaker	104	18.106	5.02
Single Mother X Worker X Homemaker	12	22.667	5.015

0, role not occupied; 1, role occupied.

Our analysis revealed that superwomen as defined by the fourth quartile of role engagement, reported similar levels of perceived stress (Mean = 19.237) than all other women (Mean = 18.659). Superwomen (Mean = 2.533) and non-superwomen (Mean = 2.517) exhibited similar levels of life satisfaction (t = 0.158, P = 0.874). We found a significant difference in network diversity (t = 3.529, P<0.001) between superwomen (Mean = 6.56) and all other women (Mean = 5.76). There was not, however, a significant difference between these two groupings in network size (t = 1.113, P = 0.267). Interestingly, superwomen as defined by those engaged in the wife/worker/mother/homemaker role combination reported the lowest perceived stress (Mean = 18.106) when compared to the distribution of stress across different roles and role combinations (presented in Table 4).

Mean life satisfaction scores for the seven roles, and various role combinations are presented in Table 5. The role with the highest mean life satisfaction was wife (N = 184, Mean = 2.663). The role of caregiver exhibited the lowest life satisfaction (N = 53, Mean = 2.339) among our seven primary roles. The difference between these two roles in life satisfaction was significant (t = 2.764, P = 0.006). We also explored the distribution of life satisfaction across role groupings that included the mother role. Single mothers (N = 26, Mean = 2.192) reported significantly (t = −2.800, P = 0.006) lower life satisfaction than mothers with partners (N = 126, Mean = 2.651). Homemakers reported significantly less life satisfaction than non-homemakers (t = 2.963, P = 0.003). The single mother/worker/homemaker combination had the lowest reported life satisfaction of all other mother combinations (N = 12, Mean = 2.00).

**Table 5:** Levels of life satisfaction for each of the seven roles and, and role combinations used in the study.

Role/Role Combination	N	Mean	SD
			
Wife 0	123	2.309	0.748
Wife 1	184	2.663	0.743
		t = 4.079, P<0.0001	
Mother 0	155	2.471	0.75
Mother 1	152	2.572	0.777
		t = 1.163, P = 0.246	
Caregiver 0	254	2.559	0.756
Caregiver 1	53	2.339	0.783
		t = 1.910, P = 0.057	
Student 0	197	2.503	0.767
Student 1	110	2.554	0.761
		t = 0.571, P = 0.568	
Worker 0	49	2.531	0.868
Worker 1	258	2.519	0.744
		t = 0.094, P = 0.925	
Volunteer 0	185	2.459	0.766
Volunteer 1	122	2.615	0.755
		t = 1.749, P = 0.081	
Homemaker 0	105	2.343	0.757
Homemaker 1	202	2.614	0.752
		t = 2.963, P = 0.003	
Mother with Partner	126	2.651	0.773
Single Mother	26	2.192	0.694
		t = 2.80, P = 0.006	
Mother X Homemaker	139	2.597	0.787
Mother X Worker X Homemaker	115	2.6	0.735
Single Mother X Worker	22	2.091	0.684
Mother With Partner X Worker X Homemaker	103	2.67	0.706
Single Mother X Worker X Homemaker	12	2	0.739

0, role not occupied; 1, role occupied.

### Analysis of Physiological Stress

Mean urinary cortisol (ng/ml) and hair cortisol (pg/mg) values for our biological-sampling group (N = 31) for our seven primary roles are reported in Table 6. We also included urinary and hair cortisol values for another role, single mother (i.e. mother without a partner) in this table. Mean urinary and hair cortisol values for each participant in our biological-sampling group (N = 31) are reported in Table 7. The range for urinary cortisol levels from the present study is comparable to previously reported levels in women (see [10, 13, 14, 94]). Previous studies on human hair cortisol in normal healthy individuals have reported levels ranging between 20 pg/mg and 46.1 pg/mg. In our sample of women, hair cortisol levels ranged from 39.28 pg/mg to 58.14 pg/mg [98] (also see [10, 15]). The single mother role had the highest level (58.14 pg/mg) followed by mother, (52.33 pg/mg) and caregiver (51.03 pg/mg). In our study, there was not a significant relationship between the level of role engagement and hair (r_s_ = 0.060, P = 0.746) or urinary (r_s_ = −0.175, P = 0.348) cortisol levels. Perceived stress (PSS) was not associated with cortisol levels in either hair (r_s_ = 0.127, P = 0.497) or urine (r_s_ = − 0.139, P = 0.453). Neither hair, nor mean urinary cortisol was correlated with any of our other primary variables.

**Table 6:** Mean cortisol levels by role for the biological sampling group.

Role	N	Mean Urinary Cortisol	Mean Hair Cortisol
		(ng/mL)	(pg/mg)
Wife	18	310.9	42.24
Mother	18	295.5	52.33
Caregiver	5	189.59	51.03
Student	14	343.46	39.28
Worker	20	298.47	44.77
Volunteer	10	248.61	45.13
Homemaker	20	301.61	38.55
Single Mother	5	249.65	58.14
Means	31	336.89 (SD 168.76)	43.49 (SD 30.81)

**Table 7:** Individual Participant Mean Urinary and Hair Cortisol Values.

Participant	Mean Urinary Cortisol	Hair Cortisol
	(ng/mL)	(pg/mg)
1	348.46	44.5
2	321.35	16.48
3	556.9	94.21
4	317.15	32.18
5	446.29	25.61
6	499.56	34.33
7	198.34	24
8	152.84	76.66
9	198.11	13
10	931.45	69.33
11	319	18.5
12	250.35	16.26
13	206.97	50.5
14	367.24	41
15	96.97	134
16	207.48	58.66
17	340.03	21.53
18	567.95	31.23
19	243.2	39
20	343.82	11.45
21	148.2	42.4
22	495.12	17.27
23	479.67	110
24	298.75	33.43
25	440.96	13.1
26	455.05	22.62
27	368.76	70
28	218.58	35.86
29	226.19	36
30	256.87	91.25
31	142.14	24

Mean urinary cortisol was calculated based on the total number of samples (n = 15) collected during the 30-day period.

### Debriefing Sessions

The responses to the questionnaire given during the debriefing session are summarized below. When asked, “*Have you heard of the term superwoman*?” all women (N = 14) responded “yes”. For the question “*What do you think it means to be a superwoman*?” the following six themes were noted: 1) women “doing it all” without losing energy, 2) raising moral and happy children, 3) juggling and balancing many roles simultaneously without “dropping the ball”, 4) doing housework and working outside the home, 5) having a supportive partner, and 6) overcoming stress. When asked, “*Do you consider yourself a superwoman*? *Why or why not*?” 29% (N = 4) of the women identified themselves as superwomen. The remaining 10 women (71%) did not. Reasons noted for identifying with the superwoman ideology included: 1) ability to keep up with all activities and roles, 2) ability to “stay sane”, 3) being independent, and 4) ability to manage stress. Reasons noted for women who did not identify themselves as superwomen included: 1) run out of energy, 2) do not work outside the home, 3) not “getting things done” as well as they should be, 4) do not have dependent children, 5) not exceptional at doing everything, 6) have help from spouse, and 7) recognize personal limitations.

The information collected during the debriefing sessions strongly reflects what was ascertained previously in the literature review. The responses of these women also corresponded well with popularized notions of the superwoman. The mean number of roles for those who participated in the debriefing was 3.09 roles, and falls within the third quartile of role engagement (i.e. non-superwoman category). Of the 4 women who self-identified as superwomen, 3 engaged in more than 3.75 roles. Of the 10 women that self-identified as non-superwomen, 8 engaged in fewer than 3.75 roles. These observations generally support our allocation of superwomen to the fourth quartile of role engagement (>3.75 roles), and non-superwoman to the first three quartiles (<3.75 roles). Based on the responses of those who identified themselves as superwomen, it is clear that they believe that superwoman are women who can handle stress as opposed to being overwhelmed by it. However, the results in Table 8 indicate that compared to women who self-identified as non-superwomen, self-identified superwomen had higher perceived stress than non-superwomen (PSS = 23.125 versus 15.6). Similarly, compared to self-identified non-superwomen, self-identified superwomen had higher mean hair (43.02 versus 37.648 pg/mg) and urinary cortisol (403.74 versus 372.43 ng/ml) levels. Self-identified non-superwomen had larger social networks when compared to self-identified superwomen (19 versus 17.75). Both self-identified superwomen and non-superwomen reported moderate to high life satisfaction.

**Table 8:** Results from debriefing session comparing self-identified superwomen and non-superwomen.

	# of Roles	PSS^1^	Life Satisfaction	Network Size	Urinary Cortisol	Hair Cortisol
Participant #					(ng/mL)	(pg/mg)
**Non superwomen**						
79	1	18	3	17	317.15	18
242	1.5	13.5	2.5	16	495.12	17.27
51	3.73	16.5	2	28	321.35	16.48
2	3.75	17	3.25	26	207.48	58.66
91	1	11	4	19	348.46	44.5
88	3.33	19	3.5	15	556.9	94.21
71	1	18	3	17	446.29	25.61
22	4.48	14.5	3	17	319	18.5
158	3.33	14.5	2.6	31	343.82	11.45
P3	3.4	14	3.25	4	368.76	70
Means	2.65	15.6	3.01	19	372.43	37.47
**Superwomen**						
20	5.1	25	2	16	250.35	16.26
P7	2.3	22	2.3	20	226.19	36
29	5.55	19	2.6	23	931.45	69.33
19	3.88	26.5	3	12	206.97	50.5
Means	4.21	23.13	2.48	17.75	403.74	43.02

^1^PSS = Perceived Stress Scale (PSS-10)

Greater values indicate higher levels of stress, satisfaction, or social network size.

## DISCUSSION

Our study investigated the association between engagement in multiple roles and perceived stress and life satisfaction in a small non-random sample of women in North America. The association between the number of roles women engaged in and two measures of physiological stress was also investigated. Under the Depletion Hypothesis we predicted that women engaged in more roles would 1) report higher levels of perceived stress, 2) exhibit higher levels of physiological stress, and 3) report lower life satisfaction than women in fewer roles. Under the Enrichment Hypothesis we predicted that women in more roles would 1) report lower levels of perceived stress, 2) exhibit lower levels of physiological stress, and 3) report greater life satisfaction. We did not find a significant correlation between role engagement and perceived stress. Similarly, in a subset of the participants (N = 31), there was not a significant relationship between the level of role engagement and hair or urinary cortisol levels. Although our comparison across quartiles of role engagement revealed that superwomen (i.e., 4^th^ quartile, ≥3.75 roles) reported higher levels of perceived stress than non-superwomen (1^st^ through 3^rd^ quartiles), this difference was not statistically significant, suggesting that multiple-role engagement in superwomen is not associated with significantly higher levels of stress. Superwomen in the fourth quartile of role engagement exhibited slightly higher, though non-significant, mean life satisfaction scores.

Our results are generally comparable with previous research using the PSS-10 survey instrument. For example, in our study perceived stress scores in the caregiver and wife roles were consistent with levels reported in previous studies (see [22, 34, 37, 44, 57, 99]). Our total sample of women had a mean PSS score of 18.802 (SD = 5.201), which is greater than stress levels presented in Cohen and Janicki-Deverts’ 2010 study [65] of the distribution of psychological stress in the United States. In that study, the authors assessed psychological stress in three national surveys administered in 1983, 2006, and 2009 using the PSS-10. The 2009 assessment revealed that women (N = 1032) had a mean PSS score of 16.14 (SD = 7.56) [91]. Kalra et al. [21] also found higher mean PSS-10 scores among pregnant women (Mean = 20.6, SD = 5.81).

Our multiple regression models did not identify role engagement as a significant predictor of perceived stress or life satisfaction. Role satisfaction, which was significantly correlated with both perceived stress and life satisfaction, was a significant predictor in both models. Combined, our findings suggest that role engagement does not affect perceptions of stress or life satisfaction in our sample of women. This finding is inconsistent both with the Depletion and Enrichment Hypotheses. Neither hypothesis, therefore, can be used to explain the effects of multiple-role engagement in our sample of North American women. What did emerge from our analysis is the importance of role satisfaction in our two outcome variables. Our results, similar to those presented by Reid and Hardy [61] suggest that it is not the quantity of roles that influence stress and satisfaction, but rather the quality of those roles.

The present study revealed a significant negative relationship between number of roles and exercise frequency. Though exercise participation has been studied more extensively in men than women [100, 101], the effect of women’s social roles (i.e. mother, wife, employee and homemaker) on exercise frequency has been examined (see [102, 103]). Our results are in line with previous research which has demonstrated that women who engage in female gender roles, similar to the ones examined in this paper, are less likely to exercise than women who are do not engage in those roles [102, 103]. With the exception of life and role satisfaction, exercise frequency has the highest negative correlation with stress. Our study did not find a significant correlation between life satisfaction and exercise frequency, suggesting exercise frequency may be an independent mitigator of perceived stress in women. This interpretation is supported by the result of our multiple regression analysis, which revealed a significant inverse relationship between exercise frequency and perceived stress when all other variables were considered.

The results of our study are consistent with what has been reported in the literature for sexual activity and life satisfaction. Previous studies have revealed that both men and women associate higher levels of sexual activity with higher life, relationship, mental, and sexual satisfaction (see [104–107]). This was clearly demonstrated in Brody and Costa’s [105] study of 2384 Swedish men and women (1255 men and 1129 women). In that study, sexual activity was significantly positively correlated with overall life satisfaction for women who reported greater frequency of penile-vaginal intercourse (r = 0.19; P<0.001) [105]. Interestingly, we found a stronger correlation between sex frequency and life satisfaction than their study (r_s_ = 0.343; P<0.001).

Our results are consistent with what has been presented in the literature on age and the number of roles and combined income. We found a significant correlation between number of roles and age, as well as between number of roles and combined income. Similar findings for the relationship between number of roles and age have been reported in the literature examining women’s engagement in multiple roles over the life course. Moen et al.’s [108] study of 427 women in upstate New York found that extended involvement in non-traditional roles such as paid worker and volunteer earlier in life promoted multiple role occupancy later in life. Of particular interest, the authors found that any involvement as a volunteer earlier in life was positively related to multiple roles in later adulthood. In addition, women with larger families were also more likely to occupy more roles later in life [108]. Other studies have reported that in comparison to younger women, women aged 45–49 years are engaged in more roles [109]. These women are often referred to as “caught in the middle” as they are more likely to simultaneously care for their children and parents [56, 109]. In short, our results are consistent with previous research suggesting that as women age they accumulate more roles.

Though our study revealed a strong relationship between roles and combined income, research interest in this area has focused largely on how roles are distributed in dual-income families (see [110]), something we did not address with our data collection. A study conducted by Statistics Canada in 2005 revealed that as wives brought in more income into the family the division of labor in the household became more equally distributed, though women still performed more domestic roles than men [110]. This suggests that as combined income increases women engage in fewer roles. This is somewhat in contrast to our results, which revealed that as more combined financial resources became available women took on more roles. The Statistics Canada (2005) report also revealed, however, that those women in dual income families reported being time-stressed with lower levels of work-life balance. In contrast, other research has reported that there is no relationship between the number of roles for both dual income men and women, and stress (see [36]).

### Limitations to the research

The results of our research are subject to several important limitations. The results in Table 4 suggest the number of roles (accumulation of roles) alone may not predict stress in multiple-role women. Although we also looked at stress in specific roles and role combinations, and role satisfaction as a measure of role quality, we did not examine the nature of specific roles (e.g., intensity) and stress. As such, our study was not able to directly examine what is likely an important dimension of role engagement, and the stress it may cause in multiple-role women. Similarly, our measure of social capital, the SNI, did not take into account the nature of the social relationships comprising that index. As such, some participants in the study may have had more profoundly supportive relationships than others with a comparatively higher SNI. This may diminish the potential role of the SNI in mitigating stress, or enhancing life satisfaction. This suggests that it may be important to consider both the qualitative and quantitative aspects of social capital when evaluating it as a mediator of stress in multiple-role women. However, we chose the SNI because it is a well-established and validated measure of social capital. As such, our results may be compared to those previously published, e.g. [62].

Although we included a measure of sexual activity we did not collect data on type of sexual activity performed. Research has demonstrated that some forms of sexual activity potentially contribute to higher levels of life satisfaction (i.e. penile-vaginal penetration is linked to lower stress more than oral sex, or masturbation) (see [105, 106]). Despite the lack of specification, our measure of sexual activity nonetheless reveals sex as a potential mitigator of stress. Further research into which form of sexual activity, along with the frequency of sexual activity may provide greater insight into the impact that sexual activity on stress levels in women.

Our study is potentially limited by small sample sizes for both the survey and the biological sampling components. Given the small sample sizes used in this study it is possible our results do not accurately reflect the relationship between multiple role engagement and stress, and the role of potential mitigating variables in North American women. It is important to note however, as summarized above, many of our results corresponded closely with those presented by previous researchers on the topic of perceived stress, life satisfaction, and role engagement in women.

### Future Research

Recent studies have posited that women’s stress responses may be different than those of men. Stress research has focused primarily on the “flight or fight” response to a psychological, physical or environmental threat to well-being. According to Taylor (2006) [111] women respond to stressful situations by “tending and befriending”. That is, women cope with stress by “tending” to their offspring and “befriending” (affiliating) with others. This may also partly explain why as the number of roles increased in the present study, so too did network size. The “tend and befriend” response is in contrast to the “flight or fight” response commonly thought to be innately human. The hormone that has been associated with this affiliative behaviour is oxytocin [111], which was not examined in this study. Along with cortisol, oxytocin is also released in response to stress. Oxytocin has been implicated in the seeking of affiliative contact, or befriending in response to stress, and is thus associated with the attenuation of psychological and physiological responses to stress [111]. Paradoxically however, with unsupportive social contact, oxytocin may also work to heighten psychological and physiological responses to stress [112]. Measuring oxytocin in hair and/or urine along with cortisol may provide insight into whether this hormone plays an important role in influencing women’s behavioral responses to stress. Investigating the role of oxytocin within the context of multiple-role women and stress could therefore provide greater insight into the results of the present study.

### Conclusion

Ours is the first study to examine a validated measure of perceived stress (i.e. PSS-10) and two validated measures of physiologic stress (hair and urinary cortisol) as possible correlates of multiple-role engagement for 7 distinct roles and 7 specific role combinations in women. Our study is also the first to examine the superwoman construct within the context of multiple-role engagement and stress. Role theory within the context of stress is divided into two broad groups: those that deplete lives and those that enrich lives [50]. Role-stress theory associates negative effects including increased stress as a consequence of role overload [113]. Role expansion theory however, suggests that individuals who engage in more roles are more likely to gain self-confidence and greater social integration leading to greater control and lower stress [114]. Our findings revealed that women engaged in more roles did not show significantly higher PSS values than women in fewer roles, nor did they exhibit significantly reduced or enhanced life satisfaction. Our results also revealed that compared with women engaged in fewer roles (<3.75 roles), women in more roles (>3.75 roles) had greater social networks. Ours is also the first study to explicitly define the superwoman using two measures of multiple-role engagement, the number of roles and a family/career role combination. Although “superwomen” are often portrayed as being more stressed our study revealed that they are not. Our results also revealed that they are not more or less satisfied with their lives than “non- superwomen”. Together, these findings suggest that neither the Depletion nor Enrichment Hypotheses posit meaningful frameworks within which to examine the occurrence of perceived stress and multiple-role engagement in North American women. Instead, our study suggests role quality, rather than role quantity may be the most important predictor of stress and life satisfaction in women.

The research presented here is significant because it is among the first to examine multiple dimensions of stress, and how that stress is experienced within the context of multiple-role engagement, a common occurrence among women in modern society. The results of our study also revealed that perceived stress was not correlated with hair or urinary cortisol. Thus the occurrence of physiologic stress should not be presumed based on perceived stress levels. Hair and urinary cortisol levels should be interpreted within their own right to assess physiologic responses to stress and the implication of such responses on women’s health and well-being. Our multi-method approach of combining data from validated survey instruments with biological markers is novel to the study of female identities. As our results have shown, popular and academic notions of female identities do not always conform to women’s experiences.

The findings from our study have potential social impact. For example, our results indicate that single mothers experience substantially higher levels of stress coupled with diminished life satisfaction. This suggests that single mothers may be at greater risk for adverse health outcomes including CHD and depression. As such, single mothers may need additional social support from state or community level organizations. Further research focused specifically on the experiences and needs of single mothers could, therefore, inform future efforts to develop such initiatives. Also importantly, our results highlight the importance of role quality in enhancing women’s lives. Future research that examines factors that influence role quality/satisfaction would provide greater insight in this area. We hope that the results of our study provide an opportunity for women to reflect on their own experiences of stress and the roles that may be enhancing, or depleting, their lives.

## Supporting Information

S1 File Women and Stress Survey 2012. (DOCX)
