# Supplementary material for: Stress and the Multiple-Role Woman: Taking a Closer Look at the “Superwoman”
Source: PLoS One. 2015 Mar 27;10(3):e0120952. doi: 10.1371/journal.pone.0120952 (PMC4376732; doi:10.1371/journal.pone.0120952)
Supplement: S1 File — (DOCX) [file pone.0120952.s001.docx]

S1

Women and Stress Survey 2012 (note: PSS-10 [89] and SNI [62] instruments are not included)

1. Please select your age group.

(18-23, 24-29, 30-37, 38-44, 45-54, 55-64, 65 or older)

1. Describe the level of satisfaction and fulfillment in your life

(5-point Likert Scale from 0[none] to 4[extremely high])

1. Were you born in Canada?

(Yes, No)

1. If you are a landed immigrant how many years have you lived in Canada?

(Less than one year, 1-5 years, 5-10 years, 10+ years, NA [i.e. not a landed immigrant]).

1. Please indicate where you currently live.

(Canada, United States, Australia, Europe, Other [please specify])

1. Which of the following best describes your current marital status?

(Currently married and living together, or living with someone in a martial-like [partner or common-law] relationship, in a relationship with boyfriend/girlfriend, recently divorced/separated from spouse, partner or common-law, widowed, single)

1. Do you find your role as a wife, partner/common-law fulfilling, satisfying or rewarding?

(5 point-Likert Scale from 0[never] to 4 [very often])

1. How many dependent children to you have?

(0, 1, 2, 3, 4, 5, 6, 7, 7 or more)

1. Do you find your role as a mother fulfilling, satisfying or rewarding?

(5 point-Likert Scale from 0[never] to 4 [very often])

1. Do any of your children have special needs?

(Yes, No, NA)

1. Are you a caregiver for your parents? (N/A if your parents are not living)

No, mother only, father only, both, N/A

1. Are you a caregiver for you in-laws (spouse, common-law, or partner’s parents)?

(No, mother-in-law only, father-in-law only, both, N/A)

1. Not including your children, parents, or in-laws, are you a caregiver for anyone else?

(Yes, No)

1. Do you find your role as a caregiver fulfilling, satisfying or rewarding?

(5 point-Likert Scale from 0[never] to 4 [very often])

1. Do you attend any classes (school, college, university, technical training, or adult education)?

(Yes, part-time [1-3 classes per term], Yes full-time [3 classes or more per term], No)

1. Do you find your role as a student fulfilling, satisfying or rewarding?

(5 point-Likert Scale from 0[never] to 4 [very often])

1. Do you belong to a sports team?

(Yes, No)

1. Please select your highest level of education completed.

(high school, diploma from junior college or trade school, university degree [bachelor’s], masters degree, doctorate degree [including D. Ed, JD, MD, or PhD], N/A)

1. Please select your combined income (you and your partner/spouse).

(Under $41,000 per year, $41,000 to $70,000 per year, $70,000 to $100,000 per year, $100,000 to $130,000 per year, $130,000 to $160,000 per year, over $160,000 per year)

1. Are you currently employed either full or part time?

(No, Yes [self-employed], Yes [employed by others])

21. If yes, please select your income. If you do not earn an income please select N/A.

Under $41,000 per year, $41,000 to $70,000 per year, $70,000 to $100,000 per year, $100,000 to $130,000 per year, $130,000 to $160,000 per year, over $160,000 per year)

22. If yes, on average how many hours do you work per week?

(Number of hours per week___________)

23. Please select the level of management that best describes your position at work. Select N/A if you are not employed.

(Non-management, low management, mid-management, upper management, N/A)

24. During a typical workweek, how much time do you spend traveling to and from work?

(Number of hours per week____________)

25. Do you find your role as a worker/employee fulfilling, satisfying or rewarding?

(5 point-Likert Scale from 0[never] to 4 [very often])

26. Do you travel out of town for business?

(Yes, No)

27. Are you currently involved in regular volunteer work?

(Yes, No)

28. If yes, how many hours on average do you volunteer per month? (if you are not a volunteer enter a “0”

(Number of volunteer hours per month____________)

29. Do you find your role as a volunteer fulfilling, satisfying or rewarding?

(5 point-Likert Scale from 0[never] to 4 [very often])

30. How many hours per week do you spend performing childcare and/or dependent care?

(Hours per week_______________)

31. How many hours per week do you spend in food preparation (grocery shopping/cooking)?

(Hours per week____________)

32. How many hours per week do spend cleaning your home?

(hours per week____________)

33. How many hours per week do you spend on extracurricular activities for children and/or dependents? (includes driving to and from activity locations).

(Hours per week____________)

34. In proportion to your partner, what percentage of childcare and/or dependent care are you responsible for?

(___%)

35. In proportion to your partner, what percentage of food preparation (grocery shopping/cooking) are you responsible for?

(___%)

36. In proportion to your partner, what percentage of cleaning your home are you responsible for?

(___%­­­­­­­)

37. In proportion to your partner, what percentage of extracurricular activities for children and/or dependents are you responsible for? (includes driving to and from activity locations)

(___%)

38. Do you have a nanny or in­home day care for your child/children?

(Yes, No)

39. Do you have a maid or house cleaning service?

(Yes, No)

40. Do you find your role as a homemaker fulfilling, satisfying, or rewarding?

(Yes, No)

41. Please select what best describes your level of stress during an average week.

(5-point Likert scale from none[0] to extremely high[4])

42. Do you suffer from sleep insomnia? (problems sleeping)

(5-point Likert scale from none[0] to extremely high[4])

43. On average how many days is your menstrual cycle?

(regular [roughly 28 days], irregular [changes from month to month], NA [post-menopausal])

44. Have you been diagnosed by your doctor as being "perimenopausal" (transitioning from normal menstrual periods to none at all)?

(Yes, No)

45. Do you take birth control pills?

(Yes, No)

46. Do you exercise?

(Yes, No)

47. If yes, on average how many times per week do you exercise and for approximately how many hours? (if no please type a '0' in both boxes)

(Times per week________________, Hours per week________________)

48. In the past 12 months, how many times have you visited a physician?

(times visited physician______________)

49. Have you had surgery or any other medical procedure within the past 60 days?

(Yes, No)

50. Have you ever been diagnosed with high blood pressure or hypertension?

(Yes, No)

51. Do you engage in sexual activity?

(Yes, No)

52. If yes, on average how many times per month? (If no, enter the number "0")

(Times per month______________)

53. Do you usually have weekly leisure time for yourself?

(Yes, No)

54. If yes, on average how many hours per week? (If no, enter the number "0")

(Hours per week_____________)

55. On average, how many servings of alcohol do you consume per week (1 serving = 1 can of beer/cider, 1 glass wine, 2 oz liquor)?

(Number of servings________________)

56. Do you smoke tobacco at least once per day?

(Yes, No
